# 

**Published:** 1885-01

**Authors:** 


### NEW BOOKS.

ELEMENTARY PRINCIPLES OF ELECTRO-THERAPEUTICS, for the use of physicians and students. With 135 illustrations. Prepared by C. M. Haynes, M. D. Published by the McIntosh Galvanic and Faradic Battery Co., Chicago, Ill. Price, $2.00.

In this book is conveyed to the medical man a deal of information with regard to the construction, care and application of electrical apparatus. The text is finely illustrated, rendering the somewhat complicated apparatus easy of comprehension. The articles showing where the different currents are applicable, and how applied, are short, concise, and inspire the reader with confidence to use them. It is a useful and readable book.

---

THE INTERNATIONAL ENCYCLOPÆDIA OF SURGERY.—A systematic treatise on the Theory and Practice of Surgery by authors of various nations, edited by John Ashurst, Jr., M. D., professor of clinical surgery in the University of Pennsylvania, illustrated with chromo-lithographs and wood-cuts. In six volumes, Vol. IV, William Wood & Co., New York, price, cloth, $6.

This work is a valuable one in many respects. It could not well be otherwise—in the literature thereof, with the able gentleman at the head who is doing the editing.

But, the wood-cuts—so called—are a disgrace to any work of such pretentions. For this fault in the work there seems to be no valid excuse. New York is abundantly supplied with most excellent engravers on wood and one cannot understand how the publishers managed to elude them.

---

THE STUDENTS' MANUAL OF HISTOLOGY, for the use of students, practitioners and microscopists. Third edition. Entirely rewritten; greatly enlarged, and newly illustrated. By Charles H. Stowell, M. D., F. R. M. S., professor of histology and microscopy, and in charge of the histological laboratory of the University of Michigan, Ann Arbor, Mich. Charles H. Stowell publisher. Price $2.00.

This book is precisely what its title claims for it; and in this regard, differs widely from the many attempts at book-making upon the same theme. Prof. Stowell is recognized everywhere, among microscopists, as a very capable manipulator of the microscope and a skillful preparer of tissues for microscopic examination. To his book he has conveyed his methods, so arranged, and so concisely and comprehensively worded as to make the study of histology a pleasure instead of a task. The illustrations are original and well drawn. We commend, most heartily, Prof. Stowell's book to the profession.

